# Frequency of Cognitive Impairment in Patients with Parkinson’s Disease

**DOI:** 10.7759/cureus.4733

**Published:** 2019-05-23

**Authors:** Muhammad Taimur, Mir Ali Asghar Shah, Maha Ali, Habiba D Barry, Syed Zohaib Maroof Hussain, Huma Shahzad, Amber Rizwan

**Affiliations:** 1 Internal Medicine, Dow University of Health Sciences, Karachi, PAK; 2 Internal Medicine, Jinnah Postgraduate Medical Center, Karachi, PAK; 3 Surgery, Dow University of Health Sciences, Karachi, PAK; 4 Surgery, Aga Khan University Hospital, Karachi, PAK; 5 Family Medicine, Dr. Ruth K.M. Pfau, Civil Hospital, Karachi, PAK

**Keywords:** parkinson disease, cognitive impairment, non-motor symptoms, cognitive deficits, pakistan

## Abstract

Introduction

More than its motor symptoms, cognitive impairment is being increasingly identified as a cause of worse functional outcome, morbidity and mortality, and caregiver dependence in Parkinson’s disease (PD). The aim of this study was to identify the frequency of cognitive decline and evaluate the factors associated with it.

Methods

In this cross-sectional study, 124 PD patients fulfilling the United Kingdom Parkinson’s Disease Society Brain Bank Clinical Diagnostic Criteria were included. Motor and non-motor symptoms were recorded. Disease duration, age at the time of onset, and severity of disease on Hoehn and Yahr Scale (HY scale) were recorded. Data was entered and analyzed using SPSSs v. 22.0.

Results

The ratio of men to women was 7.2:1. The mean age of the participants was 64 ± 10 years (range: 38-82 years). Rigidity (*n *= 121; 97.5%), bradykinesia (*n *= 119; 95.9%), and tremor (*n *= 11; 90.3%) were the three most common symptoms. Cognitive impairment was present in 45 (36.3%) patients. Cognitive decline was more frequent in patients of age less than 50 years at the time of disease onset (*p *< 0.00001) and in those with disease duration more than 10 years (*p *= 0.00001). Patients with longer disease duration had more severe disease (stage III or above on HY scale; *p *= 0.008).

Conclusion

Motor symptoms such as rigidity, bradykinesia, and tremor remain the most frequent clinical presentation among Pakistani Parkinson’s patients. One-third of these patients have cognitive dysfunction. Early age at the time of disease onset and longer duration of disease were associated with cognitive impairment.

## Introduction

Classically, Parkinson’s disease (PD) is a clinical triad of bradykinesia (slowness of movements), rigidity (stiffness), and localized tremor present even at rest. Other than these cardinal signs, the clinical presentation of PD is heterogeneous. It includes a varied spectrum of motor and non-motor signs and symptoms. PD is a complex, progressive, neurodegenerative disease with an individualized presentation in all patients [1].

PD is the second leading cause of age-related neurodegeneration. Globally, it is estimated that 10 million people are living with PD. The incidence increases with advancing age. Around 41/100,000 people in their fourth decade of life are living with PD; however, by age 80 years, the incidence increases to 1900/100,000 people. Four percent of Parkinson’s population is diagnosed at age less than 50 years [2].

Neurodegeneration in PD is characterized by pathogenetic fibrilization of α-synuclein protein and Lewy body formation in the substantia nigra, limbic system, and the neocortex. Neurodegenerative changes affect dopaminergic neurons in the substantia nigra, locus coeruleus - main noradrenergic site, raphe nuclei - where serotoninergic neurons are located, and nucleus basalis of Meynert, a part of the cholinergic complex in the basal forebrain. Hence, there is disruption in the natural equilibrium among the dopamine, noradrenaline, serotonin, and acetylcholine systems [3-4]. This underlying neuropathology is also supported by the emergence of cognitive deficit in patients with PD. Most of the cognitive and also motor functions are related to projections from the frontal lobes. Hence, it was suggested that cognitive deficit in PD is related to disruptions in the prefrontal cortex-basal ganglia-thalamocortical circuit [4].

Cognitive deficit presents as impairment in executive functions including, but not limited to, problems with planning, working memory, and visuospatial attention. Later, it may progress into clinically significant dementia. The onset of even mild cognitive impairment has been established as a predictor of deteriorating functional outcome [3]. With the incidence of dementia, the risk of mortality in PD patients increases by almost two folds [5-6].

Randomized control trials have shown promising outcomes with intensive cognitive training in enhancing cognitive function in patients with PD [7]. However, the key to addressing the cognitive decline in these patients is early recognition. The aim of this study is to evaluate the spectrum of clinical features in patients with PD, identify the frequency of cognitive decline, and evaluate the factors associated with cognitive decline in these patients.

## Materials and methods

In this cross-sectional study, conducted for one year (January to December 2018) in the outpatient neurology clinic of a tertiary care hospital in Karachi, all patients with diagnosed PD were invited to participate. This study was approved by the intuitional ethical committee and informed consent was taken from the patient.

All of these patients were already registered in the Parkinson database of the department and allotted a registration number. This registration number was recorded to not duplicate a patient once enrolled in this study. The diagnosis of PD was made in accordance with the United Kingdom Parkinson’s Disease Society Brain Bank Clinical Diagnostic Criteria [8]. Patients with incomplete clinical data, language barrier to communication, patients who did not agree to participate, or patients who are unable to communicate even with the help of family member or a doctor were excluded. Newly diagnosed cases (≤6 months to diagnosis) were also excluded. All included patients had two features of PD, the presence of two of the three cardinal clinical features: tremor, rigidity, and bradykinesia and response to levodopa.

All patients were routinely examined by a qualified neurologist as a part of their follow-up visits. The clinical findings updated by the neurologist in the patient files were accessed. Patient gender, current age, age at the time of diagnosis, duration of diagnosis, and all clinical symptoms were recorded. The severity of PD was evaluated on the Hoehn and Yahr Scale (HY scale) that records the severity of motor symptoms from Stage I to IV [9]. Data was processed and analyzed through SPSS for windows version 22.0 (NY, USA). Duration of diagnosis and age were recorded as continuous variables and then categorized for analysis. Mean and standard deviation (SD) was calculated for continuous variables. Frequencies and percentages were calculated for categorical variables including gender, clinical symptoms, and severity of disease. The correlation was calculated for the presence of cognitive decline with age at the time of diagnosis and the duration of diagnosis. Duration of diagnosis was also correlated with the severity of PD on HY scale. Chi-square was applied. *P*-value ≤0.05 was taken as significant.

## Results

A total of 124 patients of PD were included in this study. There were 109 (87.9%) men and 15 (12.1%) women. The ratio of men to women was 7.2:1. The mean age of the participants was 63.57 ± 10.41 years (range: 38-82 years). Men were slightly older than women (65.35 ± 8.01 years vs. 62.18 ± 5.38 years). There were 55 (44.3%) participants who were younger than 50 years at the time of onset of their disease and 69 (55.6%) participants were older than 50 years of age at that time. The mean duration of disease was 10.47 ± 3.58 years. There were 57 (45.9%) participants with the duration of disease less than 10 years and 67 (54.1%) participants with more than 10 years of living with the disease.

Rigidity (97.5%), bradykinesia (95.9%), and tremor (90.3%) were the three most common symptoms present in the patients. Other signs such as hypomania (85.4%), primitive reflexes (78.2%), monotonous speech (69.3%), problems with walking (68.5%), and fine motor movement (62.9%) were also very common. Cognitive impairment was present in 45 (36.3%) patients (Table 1).

**Table 1:** Clinical characteristics of patients with Parkinson’s disease (N = 124) * Patients may have more than one clinical feature

Clinical symptoms*	Frequency (%)
Rigidity	121 (97.5%)
Bradykinesia	119 (95.9%)
Tremor	112 (90.3%)
Hypomimia (Masked facies)	106 (85.4%)
Primitive reflexes	97 (78.2%)
Monotonous speech	86 (69.3%)
Difficulty walking	85 (68.5%)
Difficulty in fine work	78 (62.9%)
Constipation	72 (58.0%)
Bladder symptoms	67 (64.1%)
Stiffness	54 (54.0%)
Cognitive impairment/decline	45 (36.3%)
Falls	39 (31.5%)
Dysphagia	32 (25.8%)
Stooped posture	29 (23.3%)
Shuffling gait	21 (16.9%)
Micrographia	13 (10.4%)

Cognitive decline was correlated with the duration of disease and the patient’s age at the time of onset of disease. Among patients with cognitive decline, 32 (71.1%) were younger than 50 years at the time of onset of their disease and 13 (28.9%) were older than 50 years. In patients with no cognitive impairment, 23 (29.1%) were younger than 50 years at the time of onset of their disease and 56 (70.9%) were older than 50 years (*p *< 0.00001). When cognitive decline was correlated with duration of disease, it was seen that among patients with cognitive decline, 9 (20%) were diagnosed within 10 years and 36 (80%) have had this disease for more than 10 years. In patients with no cognitive impairment, 48 (60.7%) were diagnosed within 10 years and 31 (39.2%) have had this disease for more than 10 years (*p *= 0.00001; Table 2).

**Table 2:** Relationship of cognitive decline in Parkinson’s patients with age at onset of disease and duration of disease (N = 124) PD, Parkinson’s disease

Variables	PD patients with cognitive decline (n = 45)	PD patients with no cognitive decline (n = 79)	P value
Age of onset of disease			
Less than 50 years	32 (71.1%)	23 (29.1%)	<0.00001
More than 50 years	13 (28.9%)	56 (70.9%)	
Duration of disease			
Less than 10 years	9 (20%)	48 (60.7%)	0.00001
More than 10 years	36 (80%)	31 (39.2%)	

There were 51 (41.1%) patients at stage I and II and 73 (58.8%) patients at stage III and above on HY scale. Duration of disease was then correlated with the severity of PD. There were 23 (18.5%) patients with duration of disease ≤5 years; 16 (69.5%) were at Stage I and II and seven (30.5%) were at Stage III and above of PD. There were 34 (27.4%) patients with duration of disease 6-9 years; of these, 11 (32.3%) were at Stage I and II and 23 (67.6%) were at Stage III and above of PD. There were 67 (54.0%) patients with duration of disease ≥10 years; of these, 24 (35.8%) were at Stage I and II and 43 (64.2%) were at Stage III and above of PD (*p *= 0.008; Table 3).

**Table 3:** Relationship of duration of disease with the severity of Parkinson’s disease on Hoehn and Yahr Scale (N = 124)

Duration of disease	Stage I and II (n = 51)	Stage III and above (n = 73)	P value
≤5 years (n = 23)	16 (69.5%)	7 (30.5%)	0.008
6-9 years (n = 34)	11 (32.3%)	23 (67.6%)	
≥10 years (n = 67)	24 (35.8%)	43 (64.2%)	

## Discussion

The clinical spectrum of PD varies from patient to patient; however, the cardinal clinical features such as rigidity, tremor, and bradykinesia remain the most commonly encountered ones. Cognitive impairment was seen in more than one-third of the patients. Cognitive decline was associated with early age of onset of the disease and longer disease duration. Longer disease duration was also associated with higher severity of the disease on the HY scale.

As with other literature, there were more men with PD in this study too [10-11]. In a Japanese study, female gender was more commonly associated with PD [12]. The mean age of participants in this study was 63.57 ± 10.41 years. Comparable mean ages have been reported in local (57-64.5 years) as well as international (71; range: 67-75 years) studies [11,13-14].

Among the common clinical signs of PD in this study, rigidity, bradykinesia, and tremor were the most frequent. Similar results have been seen in other studies too. In some studies, non-motor symptoms have been more commonly reported [10,15]. In this study, 63% patients with PD showed signs of cognitive impairment. In a Nigerian study, 21% of PD patients have been reported to have cognitive deficits as compared to only 4% of control population (*p *= 0.008). However, they reported older age of onset as the independent predictor of cognitive impairment, which is contradictory to this study where lower age at the time of onset was associated with cognitive impairment [16]. In another recent study with 100 PD patients, advancing age and tremor as the dominant symptom at the onset of disease were independently correlated with poor cognitive function [17]. Among Pakistani patients of PD, cognitive deficit has been previously reported to be in 19% patients which is almost half of those as reported in this study [18-19]. In another recent Indian study, PD patients with disease duration > 10 years scored lowest on Scales for Outcomes in PD Cognition (SCOPA-COG) as compared to those with one to five and six to nine years of disease [20]. Their results of advancing cognitive impairment with increasing duration were not statistically significant, however, ours were.

The major limitation of this study was the lack of any clinical or neurological follow-up. There was no neuropsychological testing done via validated instruments. Diagnosis of cognitive impairment was made by consultant neurologists and can be subjected to human error study did not identify is study has its shortcomings too. Although, this center is the largest neurological care center in the province and deals with huge chunks of patients; it is still a single-center study. It also did not evaluate the subtype of cognitive deficit and whether or not dementia had ensued. Further cohort studies with a population-based sample must be initiated to evaluate cognitive function status in patients with PD and form the basis to new therapeutic options for delaying dementia in these patients.

## Conclusions

The present study revealed that motor symptoms such as rigidity, bradykinesia, and tremor remain the most frequent clinical presentation among Pakistani Parkinson’s patients. One-third of these patients have cognitive dysfunction. Early age at the time of disease onset and longer duration of disease were associated with cognitive impairment. However, a population-based cohort with longer follow-up will provide a more accurate picture of the factors predicting cognitive impairment in patients with Parkinson’s.

## References

[1] von Coelln R, Shulman LM (2016). Clinical subtypes and genetic heterogeneity: of lumping and splitting in Parkinson disease. Curr Opin Neurol.

[2] (2019). Parkinson’s Disease Statistics. https://parkinsonsnewstoday.com/parkinsons-disease-statistics/.

[3] Kehagia AA, Barker RA, Robbins TW (2010). Neuropsychological and clinical heterogeneity of cognitive impairment and dementia in patients with Parkinson's disease. Lancet Neurol.

[4] Solari N, Bonito-Oliva A, Fisone G, Brambilla R (2013). Understanding cognitive deficits in Parkinson’s disease: lessons from preclinical animal models. Learn Mem.

[5] Hughes TA, Ross HF, Mindham RH, Spokes EG (2004). Mortality in Parkinson's disease and its association with dementia and depression. Acta Neurol Scand.

[6] Levy G, Tang MX, Louis ED (2002). The association of incident dementia with mortality in PD. Neurology.

[7] París AP, Saleta HG, de la Cruz Crespo Maraver M (2011). Blind randomized controlled study of the efficacy of cognitive training in Parkinson's disease. Mov Disord.

[8] Hughes AJ, Daniel SE, Kilford L (1992). Accuracy of clinical diagnosis of idiopathic Parkinson’s disease: a clinico-pathological study of 100 cases. J Neurol Neurosurg Psychiatry.

[9] Goetz CG, Poewe W, Rascol O (2004). Movement Disorder Society Task Force report on the Hoehn and Yahr staging scale: status and recommendations the Movement Disorder Society Task Force on rating scales for Parkinson's disease. Mov Disord.

[10] Assadeck H, Daouda MT, Djibo FH, Maiga DD, Omar EA (2018). Clinical profile of Parkinson's disease: Experience of Niger. J Neurosci Rural Pract.

[11] Mukhtar S, Imran R, Zaheer M, Tariq H (2018). Frequency of non-motor symptoms in Parkinson’s disease presenting to tertiary care centre in Pakistan: an observational, cross-sectional study. BMJ Open.

[12] Osaki Y, Morita Y, Kuwahara T, Miyano I, Doi Y (2011). Prevalence of Parkinson’s disease and atypical parkinsonian syndromes in a rural Japanese district. Acta Neurol Scand.

[13] Ahmad I, bin Zubair US (2016). Frequency of non-motor symptoms among patients of Parkinson’s disease in Pakistan. J Pak Psych Soc.

[14] Armstrong MJ, Naglie G, Duff-Canning S (2012). Roles of education and IQ in cognitive reserve in Parkinson’s disease-mild cognitive impairment. Dement Geriatr Cogn Disord Extra.

[15] Femi OL, Ibrahim A, Aliyu S (2012). Clinical profile of parkinsonian disorders in the tropics: experience at Kano, northwestern Nigeria. J Neurosci Rural Pract.

[16] Akinyemi RO, Okubadejo NN, Akinyemi JO, Owolabi MO, Owolabi LF, Ogunniyi A (2008). Cognitive dysfunction in Nigerians with Parkinson's disease. Mov Disord.

[17] Vingerhoets G, Verleden S, Santens P, Miatton M, De Reuck J (2003). Predictors of cognitive impairment in advanced Parkinson’s disease. J Neurol Neurosurg Psychiatry.

[18] Hussain T, Mir AA (2015). Clinical pattern of morbidity among Pakistani patients of Parkinson's disease. J Neurol Sci.

[19] Khealani BA, Baig SM (2006). Clinical spectrum of Parkinson's disease from Pakistan. Singapore Med J.

[20] Chaudhary S, Joshi D, Pathak A, Mishra VN, Chaurasia RN, Gupta G (2018). Comparison of cognitive profile in young-and late-onset parkinson's disease patients. Ann Indian Acad Neurol.

